# Burnstock and the legacy of the inhibitory junction potential and P2Y1 receptors

**DOI:** 10.1007/s11302-020-09747-6

**Published:** 2020-10-30

**Authors:** Brian F. King

**Affiliations:** Research Department of Neuroscience, Pharmacology & Physiology (NPP), University College London (UCL), Gower Street, London, WC1E 6BT UK

**Keywords:** IJP, P2Y receptor, Smooth muscle, Enteric nervous system

## Abstract

The synaptic event called the inhibitory junction potential (IJP) was arguably one of the more important discoveries made by Burnstock and arguably one of his finer legacies. The discovery of the IJP fundamentally changed how electromechanical coupling was visualised in gastrointestinal smooth muscle. Its discovery also set in motion the search for novel inhibitory neurotransmitters in the enteric nervous system, eventually leading to proposal that ATP or a related nucleotide was a major inhibitory transmitter. The subsequent development of purinergic signalling gave impetus to expanding the classification of surface receptors for extracellular ATP, not only in the GI tract but beyond, and then led to successive phases of medicinal chemistry as the P2 receptor field developed. Ultimately, the discovery of the IJP led to the successful cloning of the first P2Y receptor (chick P2Y1) and expansion of mammalian ATP receptors into two classes: metabotropic P2Y receptors (encompassing P2Y1, P2Y2, P2Y4, P2Y6, P2Y11–14 receptors) and ionotropic P2X receptors (encompassing homomeric P2X1–P2X7 receptors). Here, the causal relationship between the IJP and P2Y1 is explored, setting out the milestones reached and achievements made by Burnstock and his colleagues.

## Introduction

Geoffrey Burnstock (1929–2020) was widely acknowledged for his pioneering work on ATP receptors. He defined ATP receptors as P2 receptors, in deference to the already established class of adenosine receptors which he would call P1 receptors. Later, he and Charles Kennedy would broaden the P2 receptors to P2X and P2Y subtypes—an expanded classification based on the pharmacological profile for each subtype. Burnstock applied Paton’s criteria for the identification of a neurotransmitter, to define purinergic signalling as the release of packaged ATP from synaptic vesicles in enteric nerve endings. Afterwards, Burnstock proposed that ATP is stored and released with other transmitter substances by the same nerve endings in all peripheral nerves. The restlessness of his mind led to the proposal of families of nucleotide-gated ion channels and nucleotide-activated GPCRs. In fulfilment of his expectations, members of the P2 receptor gene family were isolated and their encoded peptides characterised in a relatively short of period of time (1992–2001). Burnstock helped lead the effort to clone the first member of P2 receptor family: chick P2Y1 receptor. The pursuit of P2Y1 led to my scientific collaboration with Geoff, an alliance which produced 59 shared publications and lasted for a period of 25 years (1992–2017). We were united by a passion for the synaptic physiology of the peripheral nervous system. This commentary describes key steps in the discovery of the IJP and involvement of P2Y1.

### Gastrointestinal physiology

Before the coming of purinergic signalling [1, 2], an energised and successful Geoffrey Burnstock had spent more than 15 years making a significant contribution to our understanding of gastrointestinal smooth muscle motility. This early body of work began in 1953 with his postgraduate (PhD) research on the alimentary canal of the brown trout [3]; this gastrointestinal theme would continue for the remainder of his scientific career.

Burnstock contributed to the development of gastrointestinal physiology in 2 ways: through methodology and through discovery. In terms of methodology, Burnstock modified the Sucrose-Gap technique of Stämpfli and Straub [4, 5], to record the membrane potential and action potentials of the external longitudinal muscle layer of the gut, thereby shedding light on the electromechanical coupling of gastrointestinal smooth muscle. Burnstock and Straub were the first to do so, overcoming the problems of recording electrical events during the powerful and spontaneous movements of drug-stimulated longitudinal muscle of the small intestine in pike, frog, rabbit, rat, guinea-pig and cat, the rectum of trout, and guinea-pig taenia coli [6]. The Sucrose-Gap technique would be employed time and again to elucidate the electrogenic actions of a wide range of excitatory and inhibitory drugs on the gastrointestinal tract. Later, Burnstock and colleagues also made intracellular recordings from smooth muscle cells using sharp microelectrodes, to corroborate the electrical events recorded first by the Sucrose-Gap technique [7–9]. Such knowledge led to the rapid advancement of understanding the actions of transmitters, hormones and drugs on smooth muscle in a body of work contained in 84 papers, prior to his seminal paper (his 85th paper) on “ATP or a related nucleotide” as an inhibitory transmitter [1].

In terms of discovery, Burnstock’s defining contribution arguably came from the discovery of the inhibitory junction potential (IJP), a nerve-mediated hyperpolarisation of the membrane potential which preceded the relaxation of the longitudinal muscle in guinea-pig taenia coli [7–10]. In the fullness of time, it would be shown that ATP or a related nucleotide was the mediator of the IJP and contributed to the non-adrenergic, non-cholinergic relaxation (NANC relaxation) of the taenia coli [1, 2]. Burnstock applied the term NANC for TTX-sensitive nerve-mediated events that persisted when adrenergic and cholinergic responses were attenuated by the action of bretylium (or guanethidine) and atropine, respectively. The existence of NANC nerves had long been debated, mostly in the process of excitatory and inhibitory neurotransmission in the CNS [11–13]. There was even measured support for ATP as a transmitter from the foremost CNS neuroscientists, as best exemplified by Krnjević in 1974: “Although it may seem wasteful to use a high-energy compound like ATP as a neurotransmitter, several features would make it very suitable for such a function” [13].

The purinergic IJP in gastrointestinal smooth muscle cells showed a latency of 100 milliseconds and more from the point of stimulation of NANC inhibitory nerves to the onset of the hyperpolarisation of the membrane potential caused by the opening of potassium channels; this latency was too long to be explained by the slowest conduction velocity of unmyelinated C-fibre nerves in the GI tract [7–10]. Instead, this unexplained long latency presaged the involvement of a metabotropic receptor, with ATP first activating a surface receptor to produce an intracellular signalling molecule and cause the downstream opening of potassium channels. In the fullness of time, it would be shown that ATP acts through Gq-coupled P2Y1 receptors, leading to IP_3_-mediated calcium release, opening of small-conductance, calcium-activated potassium channels (SK channels, which are blocked by the bee-sting venom apamin), a resultant outward potassium current (*I*_*K*(Ca)_) which hyperpolarises the membrane potential of smooth muscle cells and, in turn, closes voltage-gated calcium channels to reduce free calcium levels in the intracellular fluid of smooth muscle, and finally reduces the contractility of smooth muscle [reviewed in: 14–17]. The purinergic IJP, identified first in the taenia coli of the caecum, has been demonstrated in the smooth muscle layers of mammalian stomach, small intestine, and large intestine [14–17].

### Inhibitory junction potentials (IJPs)

**Fig. 1:**
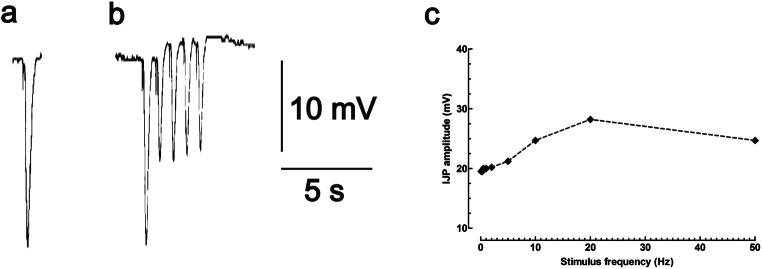
The purinergic IJP (recorded by using an intracellular microelectrode) is a rapid hyperpolarisation of the membrane potential of a smooth muscle cells (*E*_*m*_ = − 56 mV); in this case, in the circular layer of the muscularis extrema of the guinea-pig ileum. **a** The recorded electrical event is short in duration (1 s) but large in amplitude (20 mV) in response to single electrical shocks applied by field stimulation of the intrinsic inhibitory nerves. **b** Repetitive stimulation of the intrinsic inhibitory nerves (1 Hz, 5 pulses) leads to rundown of the IJP-amplitude, a phenomenon associated with the availability of primed storage vesicles at the active zone of the nerve varicosities where transmitters are released. **c** IJPs do summate if the interval between successive IJPs is sufficiently brief (100 ms at a stimulus frequency of 10 Hz, 5 pulses). The maximum amplitude of the compound IJP is approximately 30 mV for intracellular recordings made from the circular muscle of the guinea-pig ileum (King: unpublished data). The maximum amplitude of hyperpolarisation evoked by extracellularly-applied ATP (100 mM) is around 30 mV in the same tissue [54]

The purinergic IJP discovered by Burnstock is remarkable for both its brevity and large amplitude, in response to single electrical shocks applied to the intrinsic inhibitory nerves of the gut. The resultant IJP lasts only 500 to 1200 ms, yet shows an amplitude of 20 mV and more (Fig. 1a). When spontaneous mechanical activity of smooth muscle is abolished by nifedipine (L-type Ca^2+^-channel blocker), the duration of the purinergic IJP is slightly longer at around 1700 ms with a latency of onset of around 150 ms, but the amplitude is no larger [18]. The time-to peak of the purinergic IJP is around 300 ms [19]. For gastrointestinal smooth muscle, the purinergic IJP represents the fastest of inhibitory events and now is routinely called the fast-JP (fIJP, f-IJP, or IJP-f). In contrast, other inhibitory neurotransmitters identified later in the enteric nervous system evoke slower inhibitory events which are now routinely called the slow-IJP (sIJP, s-IJP, or IJP-s) [14–17].

The purinergic fast-IJP does not efficiently summate upon repetitive nerve stimulation and, instead, there is rundown in the amplitude of successive IJPs at stimulus frequencies higher than 0.1 Hz (1 pulse every 10 s) (Fig. 1b). However, there is still limited summation at higher stimulus frequencies. The purinergic fast-IJP to single electric shocks is around 70% of the maximum IJP-amplitude; a higher number of pulses or a higher frequency of pulses marginally enhances the amplitude of the resultant compound IJP (Fig. 1c). The maximal amplitude of the purinergic fast-IJP depends on the difference between the membrane potential of smooth muscle (*E*_*m*_) and the reversal potential for potassium channels (*E*_*K*_). Thus, the maximum amplitude of the fast-IJP is defined by the driving force (*E*_*K*_*-E*_*m*_) and is around 25–30 mV with *E*_*m*_ values of − 60 mV and *E*_*K*_ of − 85 to − 90 mV (Fig. 1c). Since single electrical shocks so efficaciously produce a fast-IJP of 20 mV in amplitude, the purinergic fast-IJP is both powerful and synaptically economical.

The purinergic fast-IJP is often accompanied by a non-purinergic slow-IJP in many smooth muscle bands of the gut [20, 21]. This is certainly true for guinea-pig taenia coli [18, 22], which Burnstock had used regularly in his studies of purinergic signalling. The nature of the slow-IJP is beyond the scope of this commentary but involves the production and release of nitric oxide (NO) from intrinsic inhibitory nerves, and activation of cGMP-dependent potassium channels in smooth muscle. Notably, the slow-IJP summates with increasing numbers of stimulus pulses or increasing stimulus frequency. Both ATP and NO are considered to be co-released by the intrinsic inhibitory nerves of the gut. There is co-localisation of ATP-containing storage vesicles and nNOSα in nerve varicosities harvested from the myenteric plexus; their presence in synaptic varicosities is dependent on intra-axonal kinesin and myosin motors in intrinsic inhibitory nerves [23, 24]. Accordingly, purinergic and nitrergic transmission in the gut are impaired in mice deficient in the transport motor, myosin Va [23, 24]. Neuronal nitric oxide synthase (nNOS) is now used as a marker in intrinsic inhibitory nerves for the co-localisation of ATP, NO and yet another peptidergic inhibitory transmitter responsible for a prolonged-IJP (which involves either PACAP or VIP) [15]. These intrinsic inhibitory nerves are classed phenotypically as ATP/NOS/VIP/PACAP/ENK (short anal projection) and ATP/NOS/VIP/PACAP/GRP (long anal projection) for descending inhibitory pathways to the circular muscle of the guinea-pig ileum [15]. It has been postulated that purinergic inhibition is concerned more with descending inhibition in the peristaltic reflex, whereas nitrergic inhibition may determine the general degree of excitability and contractility of smooth muscle [25]. To this end, there is a decreased rate in P2Y1 knockout mice of the colonic transit of faecal pellets which is also observed in WT-mice treated with the selective P2Y1 antagonist, MRS2500 [21]. On the other hand, the impairment of nitrergic transmission caused by polymorphisms of the NOS-1 gene is strongly associated with oesophageal muscle spasm (achalasia) in human infants [26].

### Metabotropic P2Y receptors

**Fig. 2:**
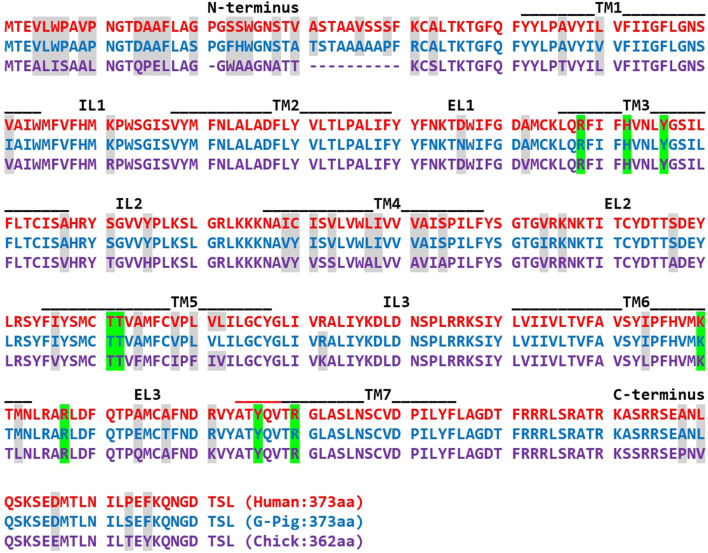
The amino acid sequence of P2Y1 orthologues (human P2Y1, red; guinea-pig P2Y1, blue; chick P2Y1, purple). The location of the α-helices forming the transmembrane spanning domains (TM1-TM7) are shown for the hP2Y1 crystal [40]. Extracellular loop (EL1–3) and intracellular loops (IL1–3) and N- and C-termini are shown too. P2Y sequences were aligned using *Clustal Omega* and anchored to the amino acid sequence for TM1 of hP2Y1. Human and guinea-pig P2Y1 peptides are 373 amino acids in length, whereas the N-terminus of chick P2Y1 is shorter by 11 amino acids and the resultant peptide is 362 amino acids in length. Guinea-pig P2Y1 peptide is 95% identical to human P2Y1, whereas chick P2Y1 is 83% identical to human P2Y1. Non-conserved amino acids are marked by grey shading. The amino acid residues marked by green shading are predicted to interact with the agonist 2-MeSADP by Molecular Dynamics modelling of the human P2Y1 crystal. These residues are conserved for the three P2Y1 orthologues and are predicted to form the binding pocket for 2-MeSADP [41]

Burnstock proposed the existence of extended families of metabotropic and ionotropic ATP receptors [27], based on the discovery of two cloned P2Y receptors now named P2Y1 and P2Y2 [28, 29] and two cloned P2X receptors now named P2X1 and P2X2 [30, 31]. The numbering system developed by Burnstock for these extended families of cloned P2 receptors is summarised elsewhere, yet represents a fundamental element of housekeeping in the P2 receptor field [32]. The cloned P2Y receptor proteins are 328–377 amino acids in length and are some of the shorter GPCRs found in mammalian cells. They possess seven hydrophobic regions, forming the trans-membrane spanning regions TM1–TM7, which lie between an extracellular N-terminus (27–51 residues in length) and cytosolic C-terminus (15–68 residues in length), with consensus motifs for intracellular kinases on the intracellular loops and cytosolic C-terminus (Fig. 2). Alignment of the protein sequences for the TM1–TM7 region reveals 17–62% identity (35–80% similarity) [33].

Burnstock helped lead the effort to clone ATP receptors. The first recombinant ATP receptor was chick P2Y1 (cP2Y1), isolated as clone 803 from a cDNA library of chick brain screened for homologous sequences to a partial guinea-pig adenosine-like sequence (RDC1) [28]. When the chick is close to hatching, its brain undergoes a rapid phase of expression of receptor mRNAs; accordingly, the hatched chick is a highly precocious neonate. Impressively high levels of P2Y1 receptor expression were observed for [^35^S]-dATPαS radioligand binding in chick brain (37 pmol/mg protein for cP2Y1 versus 1–2 pmol/mg protein for muscimol binding at GABA_A_) [28, 34]. Transcripts for chick P2Y1 are not only localised in neurons and astrocytes of the brain; its mRNA is present in spinal cord, gastrointestinal tract, spleen, and leg muscle in adult chicken [28]. A remarkably high level of P2Y1 protein expression also was observed in neurons in the hippocampus of human brain [35], with a wide distribution of human P2Y1 (hP2Y1) in many other neuronal and non-neuronal peripheral tissues [36]. A full description of hP2Y1 expression and distribution appears online in the *IUPHAR* P2Y1 database [37].

**Fig. 3:**
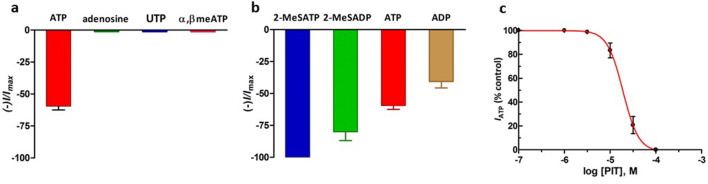
Pharmacological profile of cP2Y1 expressed in *Xenopus laevis* oocytes. **a** Chick ATP receptor was defined as a P2 receptor and not a P1 receptor, because it was activated by ATP but not by adenosine. The activity of ATP but not UTP and α,βmeATP defined a P2Y receptor and excluded the involvement of P2U or P2X receptors. **b** Chick ATP receptor was activated potently by 2-MesATP and 2-MeSADP which were full agonists, whereas ATP and ADP were partial agonists. EC_50_ values were: 2-MeSATP, 10 ± 1 nM; ATP, 155 ± 50 nM; ADP, 258 ± 40 nM. Nucleotide responses were inward chloride currents (*V*_*h*_, − 40 mV) and all agonists were tested at 1 μM for 60 s, every 30 min. **c** ATP responses at the chick P2Y receptor were antagonised in a nonsurmountable way by 2,2-Pyridylisatogen Tosylate (PIT, 0.1–100 μM), with an IC_50_ value of 13.2 ± 9 μM [44]. PIT blocks ATP responses, but not adenosine responses, in the guinea-pig taenia coli [45]

The pharmacological and operational profiles have been studied for chick P2Y1 expressed heterologously in *Xenopus laevis* oocytes, where agonist responses evoke a calcium-dependent chloride current (*I*_Cl(Ca)_) [28, 38]. ATP and not adenosine activated the chick ATP receptor, thus defining a P2 receptor (Fig. 3a). The reversal potential of ATP-evoked currents was around − 25 mV, implicating the involvement of a chloride current. Substitution of extracellular calcium ions with extracellular barium ions reduced the amplitude of *I*_Cl(Ca)_ evoked by either hyperpolarising voltage steps or extracellular ATP [28]. Chick P2Y1 showed an agonist potency order reminiscent of the mammalian native P2Y receptor with 2-MeSATP>2-MeSADP>ATP > ADP as agonists, while UTP, α,βmeATP and BzATP were inactive (Fig. 3a,b). When the chick P2 receptor was expressed transiently in COS-7 cells, ATP receptor activation led to a suramin-sensitive increase in levels of intracellular inositol triphosphate (IP_3_) [38]. The binding of the radioligand [^35^S]-dATPαS was displaced by 2-MeSATP>ATP > ADP at high affinity, whereas adenosine, β,γmeATP and UTP did not [38]. The non-selective P2 receptors antagonists, suramin and Reactive Blue-2, reversibly antagonised evoked ATP-responses as either *I*_Cl(Ca)_ or raised IP_3_ levels at chick P2Y1 [38]. Thus, pharmacological, biochemical and structural data defined the chick P2Y1 receptor as the first metabotropic P2Y receptor. Over 280 species orthologues of cP2Y1 have been cloned, including human P2Y1 where ADP and 2-MesADP are more potent agonists than ATP and 2-MeSATP [39]. The predicted binding site for 2-MeSADP has been elucidated by molecular dynamics modelling of the known human P2Y1 crystal [40, 41].

### Purinergic IJP and P2Y1receptors

Based on the pharmacological profiling of native P2Y receptors [42], Burnstock proposed that cP2Y1 was pharmacologically related to the ATP-activated P2Y receptor in guinea-pig taenia coli [43]. Moreover, pyridylisatogen tosylate (PIT) was found to be a nonsurmountable antagonist at cP2Y1 (Fig. 3c) [44], with PIT also known to block ATP relaxations but not adenosine relaxations in the guinea-pig taenia coli [45]. Later, PIT would be shown to be a selective antagonist for human P2Y1 receptors and not human P2Y2, P2Y4, P2Y6, P2Y11 or P2Y12 [46]. The guinea-pig P2Y1 (gpP2Y1) receptor has since been cloned and characterised by Wood and colleagues, with an agonist potency order of 2-MeSADP> 2-MeSATP>ADP>ATP for gpP2Y1-evoked calcium signals in HEK293 cells [47]. PIT was not tested on gpP2Y1, but the P2Y1-selective antagonist MRS2179 was highly effective at blocking this recombinant P2Y receptor [47].

The link between P2Y1 and the purinergic fast-IJP was better forged after the development by Jacobson of the deoxyadenosine bisphosphate derivatives (including MRS2179, MRS2279 and MRS2500) as highly-selective P2Y1 antagonists [48–50]. Burnstock did not play a major role in screening bisphosphate compounds as P2Y1 receptor antagonists; instead, our laboratory carried out complementary tests on these bisphosphates for activity at recombinant P2X receptors [51]. The blocking activity of commercially available MRS2179 was first confirmed for the fast-IJP of the circular and longitudinal muscle layers of the human colon [52]. Later, the blocking activity of MRS2279 and MRS2500 was confirmed for the purinergic fast-IJP of the circular muscle layer of the human colon [53]. Ultimately, the involvement of P2Y1 in the purinergic fast-IJP was solidified using P2Y1-null mice and by cross-comparison of knockout data from WT-mice treated with selective P2Y1 antagonists [20, 21].

## Summary

The early discovery of the IJP by Burnstock was a potent stimulus for wide ranging research over many successive decades. It caused us to reflect on how neurotransmitters operate potassium channels and which neurotransmitters other than acetylcholine and noradrenaline could do so in the peripheral nervous system. It led to the proposal that ATP itself is a transmitter. In turn, it caused us to study receptor mechanisms and explore the attendant medicinal chemistry of ATP receptors. It led to studies on the molecular signalling of ATP receptors and structure of ATP receptors. Cloning of chick P2Y1 led to homology screening and discovery of a broad family of P2Y receptors, the distribution of which led to wider research on the fundaments of cell physiology and experimental medicine. It gave rise to further medicinal chemistry for P2Y subtype-selective agonists and antagonists as potential therapeutics for human diseases. This enduring legacy has served science well for almost 6 decades; it will be interesting to see how farther the Burnstock legacy will take us.

## Abbreviations

ENK: Enkephalin
GRP: Gastrin-releasing peptide
GPCR: G protein-coupled receptor
MRS2179: 2′-Deoxy-N^6^-methyladenosine 3′,5′-bisphosphate
MRS2279: 2-Chloro N^6^-methyl-(N)-methanocarba-2′-deoxyadenosine-3′,5′-bisphosphate
MRS2500: 2-iodo-N^6^-methyl-(N)-methanocarba-2′-deoxyadenosine-3′,5′-bisphosphate
NOS: Nitric oxide synthase
PACAP: Pituitary adenylate-cyclase activating peptide
PIT: 2,2-Pyridylisatogen Tosylate
SK channels: Small-conductance Ca^2+^-activated K^+^-channels
TTX: Tetrodotoxin
VIP: Vasoactive intestinal polypeptide
